# The effects of low tidal ventilation on lung strain correlate with respiratory system compliance

**DOI:** 10.1186/s13054-017-1600-x

**Published:** 2017-02-03

**Authors:** Jianfeng Xie, Fang Jin, Chun Pan, Songqiao Liu, Ling Liu, Jingyuan Xu, Yi Yang, Haibo Qiu

**Affiliations:** Department of Critical Care Medicine, Nanjing ZhongDa Hospital, School of Medicine, Southeast University, 87 Dingjiaqiao Road, Gulou District, Nanjing, Jiangsu 210009 China

**Keywords:** Acute respiratory distress syndrome, Mechanical ventilation, Tidal volume, Lung strain, Ventilator-induced lung injury

## Abstract

**Background:**

The effect of alterations in tidal volume on mortality of acute respiratory distress syndrome (ARDS) is determined by respiratory system compliance. We aimed to investigate the effects of different tidal volumes on lung strain in ARDS patients who had various levels of respiratory system compliance.

**Methods:**

Nineteen patients were divided into high (C_high_ group) and low (C_low_ group) respiratory system compliance groups based on their respiratory system compliance values. We defined compliance ≥0.6 ml/(cmH_2_O/kg) as C_high_ and compliance <0.6 ml/(cmH_2_O/kg) as C_low_. End-expiratory lung volumes (EELV) at various tidal volumes were measured by nitrogen wash-in/washout. Lung strain was calculated as the ratio between tidal volume and EELV. The primary outcome was that lung strain is a function of tidal volume in patients with various levels of respiratory system compliance.

**Results:**

The mean baseline EELV, strain and respiratory system compliance values were 1873 ml, 0.31 and 0.65 ml/(cmH_2_O/kg), respectively; differences in all of these parameters were statistically significant between the two groups. For all participants, a positive correlation was found between the respiratory system compliance and EELV (R = 0.488, *p* = 0.034). Driving pressure and strain increased together as the tidal volume increased from 6 ml/kg predicted body weight (PBW) to 12 ml/kg PBW. Compared to the C_high_ ARDS patients, the driving pressure was significantly higher in the C_low_ patients at each tidal volume. Similar effects of lung strain were found for tidal volumes of 6 and 8 ml/kg PBW. The “lung injury” limits for driving pressure and lung strain were much easier to exceed with increases in the tidal volume in C_low_ patients.

**Conclusions:**

Respiratory system compliance affected the relationships between tidal volume and driving pressure and lung strain in ARDS patients. These results showed that increasing tidal volume induced lung injury more easily in patients with low respiratory system compliance.

**Trial registration:**

Clinicaltrials.gov identifier NCT01864668, Registered 21 May 2013.

**Electronic supplementary material:**

The online version of this article (doi:10.1186/s13054-017-1600-x) contains supplementary material, which is available to authorized users.

## Background

Mechanical ventilation is an established intervention in the supportive management of patients with acute respiratory distress syndrome (ARDS) [1]. Low tidal volume (V_T_) ventilation has been demonstrated to decrease mortality in ARDS patients and has become the standard ventilation strategy in practice [2, 3]. However, ARDS mortality remains high and is associated with ventilator-induced lung injury (VILI), even when low tidal volume ventilation is used [4]. In addition, the effect of low tidal volume on ARDS mortality is determined by respiratory system compliance [5–7]. Deans and colleagues reanalyzed the ARDSNet Trial data and found that decreasing the tidal volume can significantly reduce mortality in patients whose respiratory system compliance is < 0.6 ml/(cmH_2_O/kg) but it increases mortality in patients whose respiratory system compliance is ≥ 0.6 ml/(cmH_2_O/kg) [5].

Lung strain, which is the ratio of the change in lung volume during respiration to the resting lung volume, directly reflects changes in lung tissue mechanics and is associated with VILI [8]. Protti et al. found that there was a safe lung strain threshold during mechanical ventilation [9]. Non-physiological strain has been proposed to be one of the key mechanisms of VILI [8–10]. A recent study reported that patients with higher strain values showed fourfold increases in the interleukin (IL)-6 and IL-8 concentrations in their bronchoalveolar lavage fluid that were associated with aggravated lung injury [11]. In recent years, strain has been applied to titrate the positive end-expiratory pressure (PEEP) and tidal volume settings [12, 13].

However, in ARDS patients, in whom the respiratory system compliance varies, the strain differs among patients even under the same tidal volume ventilation conditions. Caironi et al. demonstrated that the strain was significantly greater in ARDS patients with higher amounts of recruitable lung compared with those with lower amounts of recruitable lung under similar tidal volume application conditions [12]. In addition, ARDS patients with higher amounts of recruitable lung had much more severe disease and had relative low respiratory system compliance [14]. Therefore, in ARDS patients with more baby lung, which has, to some extent, higher respiratory system compliance, strain may not increase significantly and may remain below the safe threshold for inducing VILI despite increases in the tidal volume [15]. However, low tidal volume ventilation increases the risk for supplementary sedation, curarization, and atelectasis. Therefore, setting strain-guided individual tidal volumes may be a rational approach [16].

It is important to use low tidal volumes in the appropriate patients to avoid the side effects of this intervention. We hypothesized that the risk of exceeding a safe level of strain is lower in the patients with greater respiratory system compliance. Therefore, we performed this study to evaluate the effects of various tidal volumes on lung strain in ARDS patients with different levels of respiratory system compliance with the goal of optimizing the individual tidal volume settings. The primary outcome of our study was that lung strain is a function of the tidal volume in patients with different levels of respiratory system compliance. Some of the results of this study have been previously reported in the form of an abstract [17].

## Methods

### Patients

Patients diagnosed with ARDS according to the Berlin definition who had received invasive mechanical ventilation were selected for inclusion in this study. The exclusion criteria were age below 18 or above 85 years old, fraction of inspired oxygen (FiO_2_) greater than 90%, hemodynamic instability that did not respond to vasoactive drugs, history of chronic pulmonary disease, pregnancy or the presence of an airway leak. All patients received analgesia and sedation at levels selected by the intensivists.

This prospective observational study was performed in a university-affiliated hospital. The protocol was approved by the Institutional Ethics Committee of ZhongDa Hospital (approval number 2013ZDSYLL074.0) and registered on clinicaltrials.gov (registration no. NCT01864668). Each patient (or designated proxy) provided written informed consent. Participation in the study did not necessitate any changes in treatment.

### Study design

After enrollment, the patients were sedated and placed in the supine position, after which they were subjected to volume-controlled mechanical ventilation. With the exception of tidal volume, the ventilation parameters that had been set by the attending physicians were not changed during the study. PEEP and FiO_2_ were set according to the ARDSNet PEEP/FiO_2_ table (low PEEP strategy) [2]. The end-expiration lung volumes (EELV) were measured using an oxygen washin/washout technique at tidal volumes of 6, 8, 10, and 12 ml/kg predicted body weight (PBW). Tidal volume was not increased if the plateau was higher than 30 cm H_2_O. The trial was terminated when patients met the following conditions: heart rate > 140 beats/min or varying by ≥ 20%; SpO_2_ < 88%; or systolic blood pressure > 180 mmHg or < 90 mmHg.

### Data collection and definition

We recorded the patient characteristics after inclusion. Respiratory system compliance was calculated as the ratio between tidal volume and driving pressure (plateau pressure minus PEEP) at baseline. The patients were divided into high (C_high_ group) and low respiratory system compliance (C_low_ group) groups based on their respiratory system compliance values [6]. We defined compliance ≥ 0.6 ml/ (cmH_2_O/kg) as C_high_ and compliance < 0.6 ml/ (cmH_2_O/kg) as C_low_. Lung strain was computed as the ratio between the tidal volume and EELV. Lung strain ≥ 0.27 indicates VILI according to a recent study [11].

### Measurement of EELV

EELV was measured using an oxygen washin/washout technique, and the results were calculated by estimating the change in the nitrogen volume after a 10% increase in FiO_2_. A second EELV measurement was obtained after returning FiO_2_ to the baseline. These values were subsequently averaged [18, 19]. The various tidal volumes were applied in a randomized order (V_T_ = 6 ml/kg, 8 ml/kg, 10 ml/kg, or 12 ml/kg), and no other settings were changed. EELV was measured after stabilization using the functional residual capacity (FRC) INview function of the GE ventilator (GE Engström Carestation, GE Healthcare, Chicago, IL, USA) at the various tidal volumes.

### Statistical methods

The data are presented as the means ± standard deviation ($\overline{\mathrm{x}}$ ± s) for continuous variables, and the frequency and percentage in each category for categorical variables. The continuous variables were compared using a two-sample *t* test. The categorical variables were compared using the chi-square test. Correlations between continuous variables were computed using the Pearson coefficient. For ordinal categorical data, the Spearman correlation coefficient was used. SPSS 16.0 (SPSS Inc., Chicago, IL, USA) and Graphpad Prism5 software (GraphPad Software, La Jolla, CA, USA) were used for the statistical analysis and graphing. Missing values in EELV were imputed using the hot-deck imputation method [19]. A *p* value lower than 0.05 was considered statistically significant.

## Results

### Baseline characteristics of patients

**Fig. 1:**
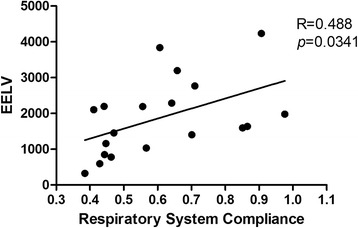
The correlation between compliance and EELV values during mechanical ventilation at the basic tidal volume setting in all included patients. *Abbreviations*: *EELV* end-expiratory lung volume

Nineteen ARDS patients were included in this study, nine of whom met the high compliance criteria. Table 1 shows the characteristics of the patients. The ARDS severity was categorized according to the Berlin Definition [20]. The PEEP and tidal volume settings did not differ between the two groups. However, EELV and strain were significantly different (*p* = 0.005 and *p* = 0.007, respectively, Table 1). A positive correlation was found between respiratory system compliance and EELV (R = 0.488, *p* = 0.034) (Fig. 1).

**Table 1:** Baseline demographic and clinical data of the patients ($\overline{\mathrm{x}}$ ±s, *n* = 19)

	All (*n* = 19)	C ≥ 0.6 (*n* = 9)	C < 0.6 (*n* = 10)	*p* value
Male n,%	13 (68)	7 (78)	6 (60)	0.628
Age, years	70 ± 14	71 ± 12	68 ± 17	0.687
Height, cm	170 ± 6	170 ± 7	170 ± 7	0.937
IBW, kg	64.9 ± 7.3	65.4 ± 7.9	64.4 ± 7.0	0.769
APACHE II	19.3 ± 6.8	18.6 ± 8.3	19.9 ± 5.6	0.681
Murray score	2.4 ± 0.5	2.2 ± 0.4	2.6 ± 0.6	0.082
ARDSp/ARDSexp, n	15/4	7/2	8/2	1.000
PaO_2_/FiO_2_, mmHg	200 ± 87	212 ± 69	189 ± 103	0.580
Hours of ventilation	67.6 ± 36.1	69.8 ± 34.0	65.7 ± 39.7	0.814
V_T_, ml/kg	6.6 ± 1.1	6.7 ± 1.1	6.5 ± 1.3	0.720
PEEP, cmH_2_O	8.2 ± 1.7	8.3 ± 1.6	8.1 ± 1.9	0.777
C, ml/(cmH_2_O/kg)	0.65 ± 0.22	0.83 ± 0.20	0.49 ± 0.07^*^	0.001
EELV, ml	1873 ± 1065	2546 ± 1024	1267 ± 689^*^	0.005
Strain	0.31 ± 0.19	0.19 ± 0.08	0.42 ± 0.20^*^	0.007
Severity of ARDS, n (%)				
mild	7 (36.8)	4 (44.4)	3 (30.0)	0.650
moderate	10 (52.6)	5 (55.6)	5 (50.0)	1.000
severe	2 (10.5)	0 (0)	2 (20.0)	0.474
28-day mortality, n (%)	8 (42.1)	3 (33.3)	5 (50)	0.788

*Abbreviations*: *IBW* ideal body weight, *APACHE II* Acute Physiology and Chronic Health Evaluation II, *ARDSp/ARDSexp* ARDS with pulmonary and extrapulmonary origin, *V*_*T*_ tidal volume, *PEEP* positive-end expiratory pressure, *C* respiratory system compliance, *EELV* end-expiratory lung volume, *ARDS* acute respiratory distress syndrome

^*^ *p* < 0.05 for comparison with C ≥ 0.6 subjects

### Respiratory mechanics and hemodynamics on different tidal volume

Increases in the tidal volume were associated with corresponding increases in the peak pressure, mean airway pressure, and plateau pressure. Additionally, when V_T_ increased from 6 ml/kg PBW to 8 ml/kg, the plateau pressure increased significantly (*p* = 0.035) (Table 2). The various tidal volumes had no significant effect on hemodynamics (Additional file 1: Table S1).

**Table 2:** Respiratory mechanics for the patients during mechanical ventilation at various tidal volumes ($\overline{\mathrm{x}}$ ± s, *n* = 19)

	V_T_ (ml/kg)			
	6	8	10	12
PEEPtot, cmH_2_O				
C ≥ 0.6 (*n* = 9)	8.3 ± 1.6	8.3 ± 1.6	8.3 ± 1.6	8.1 ± 1.6
C < 0.6 (*n* = 10)	8.1 ± 1.9	8.1 ± 2.0	8.1 ± 2.0	8.1 ± 2.0
Ppeak, cmH_2_O				
C ≥ 0.6 (*n* = 9)	20.1 ± 4.4	24.4 ± 4.0	31.3 ± 5.7^*^	34.9 ± 4.9^*^
C < 0.6 (*n* = 10)	27.1 ± 3.9	31.0 ± 4.9^#^	33.9 ± 4.9^*^	35.6 ± 5.3^*^
Pplat, cmH_2_O				
C ≥ 0.6 (*n* = 9)	15.7 ± 2.5	19.3 ± 2.5^*^	22.3 ± 3.1^*^	24.4 ± 2.3^*^
C < 0.6 (*n* = 10)	21.8 ± 2.6^#^	25.6 ± 4.1^#^	26.7 ± 3.2^*#^	27.6 ± 2.6^*#^
Pmean, cmH_2_O				
C ≥ 0.6 (*n* = 9)	10.1 ± 2.5	11.1 ± 2.0	12.6 ± 2.4	13.0 ± 2.1
C < 0.6 (*n* = 10)	12.6 ± 3.5	14.3 ± 4.9	14.6 ± 4.9	14.8 ± 4.8
EELV, ml				
C ≥ 0.6 (*n* = 9)	2804 ± 1562	2717 ± 1124	2628 ± 1086	2534 ± 1048
C < 0.6 (*n* = 10)	1317 ± 775^#^	1347 ± 886^#^	1485 ± 840^#^	1482 ± 1230
C, ml/(cmH_2_O/kg)				
C ≥ 0.6 (*n* = 9)	0.85 ± 0.23	0.75 ± 0.14	0.70 ± 0.11	0.73 ± 0.10
C < 0.6 (*n* = 10)	0.45 ± 0.08^#^	0.40 ± 0.16^#^	0.48 ± 0.10^#^	0.47 ± 0.09^#^

*Abbreviations*: *V*_*T*_ tidal volume *PEEPtot* total positive-end expiratory pressure, *Ppeak* peak airway pressure, *Pplat* plateau pressure, *Pmean* mean airway pressure, *EELV* end-expiratory lung volume, *C* respiratory system compliance

^#^ *p* < 0.05 for the comparison with C ≥ 0.6 subjects

^*^ *p* <0.05 for the comparison with V_T_ = 6 ml/kg PBW

### Effect of different tidal volumes on driving pressure

**Fig. 2:**
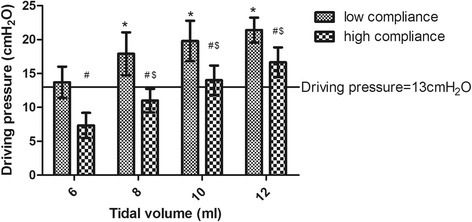
The driving pressure in the patients at each tidal volume. ^*^ *p* < 0.001 for the comparison with V_T_ = 6 ml/kg in C_high_ group. ^$^ *p* < 0.001 for the comparison with V_T_ = 6 ml/kg in the C_low_ group. ^#^ *p* < 0.001 for the comparison with the C_low_ group patients

The driving pressure significantly increased when the tidal volume was increased from 6 to 12 ml//kg PBW in both groups (Fig. 2). However, compared with those in the C_high_ group, the driving pressure was significantly higher in the patients in the C_low_ group at each tidal volume (Fig. 2). Using a driving pressure of 13 cmH_2_O as the threshold for lung injury according the work of Amato et al. [20], it was much easier to exceed the safe driving pressure while increasing the tidal volume in the patients in the C_low_ group (Fig. 2).

### Effect of different tidal volumes on strain

When the tidal volume was increased gradually from 6 ml/kg to 12 ml/kg, the mean lung strain increased gradually from 0.31 ± 0.27 to 0.52 ± 0.46. There was a strong positive correlation between the tidal volume and strain (R = 0.956, *p* < 0.001). The EELV was much higher in the patients with high respiratory system compliance than in the patients with low compliance (Additional file 2: Figure S1).

**Fig. 3:**
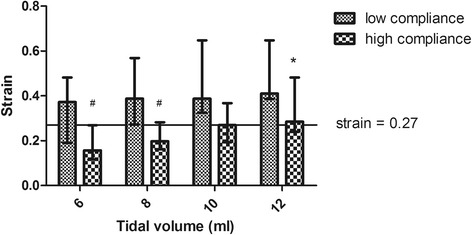
The strain in the patients at each tidal volume. ^*^ *p* < 0.05 for the comparison with the subjects at V_T_ = 6 ml/kg subjects. ^#^ *p* < 0.05 for the comparison with the C_low_ group patients

In the C_high_ group, the lung strain did not increase significantly when the tidal volume was increased from 6 to 10 ml/kg PBW. However, the strain increased significantly when the tidal volume was increased to 12 ml/kg PBW (*p* = 0.012) (Fig. 3). Interestingly, we found that the strain exceeded the safe threshold at tidal volume settings above 10 ml/kg PBW even in the patients with higher compliance.

In the C_low_ group, there were no significant differences among the strain values at volumes of 8, 10 or 12 ml/kg PBW compared with the tidal volume of 6 ml/kg PBW (Fig. 3). However, among these patients, the strain values were much higher than the corresponding values in the patients with high compliance (Fig. 3). In these patients, it was much easier to exceed “lung injury” levels of strain with increasing tidal volumes. We also found that in most patients with low compliance, the target for safe lung strain was exceeded even during ventilation with a tidal volume of 6 ml/kg PBW. Interestingly, the change in the strain associated with the increase in the tidal volume from 6 to 8 ml/kg PBW was much higher in the patients with high respiratory system compliance (*p* = 0.0002) (Additional file 3: Figure S2).

### The relationship between driving pressure and lung strain

**Fig. 4:**
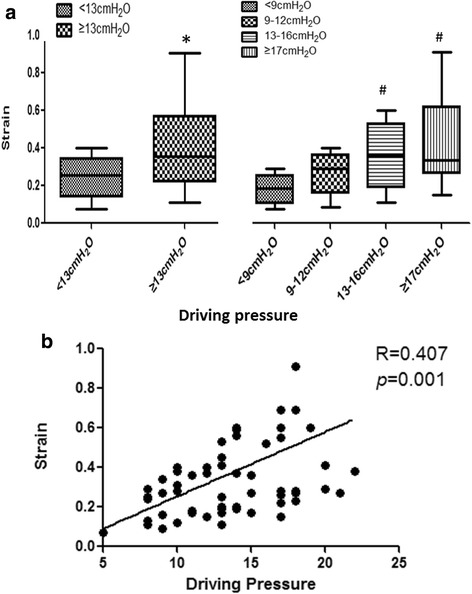
**a** The lung strain at different levels of driving pressure. **b** The correlation between strain and driving pressure. ^*^ *p* < 0.05 for the comparison with driving pressure less than 13 cmH_2_O. ^#^ *p* < 0.05 for the comparison with patients with driving pressure less than 9 cmH_2_O

We analyzed the relationship between the driving pressure and lung strain. The strain increased gradually as a function of the driving pressure. Compared to patients in whom the driving pressure was less than 9 cmH_2_O, the strain was not significantly greater in the patients with driving pressures of 9–12 cmH_2_O. However, the strain was significantly increased in the patients in whom the driving pressure was greater than 13 cmH_2_O (Fig. 4a). We also compared the differences in the strain between patients with driving pressures less than 13 cmH_2_O and those greater than or equal to or 13 cmH_2_O. The strain was determined to be significantly higher in the patients with high driving pressures (Fig. 4a). However, we found only a relatively moderate positive relationship between the driving pressure and lung strain (R = 0.407, *p* = 0.001) (Fig. 4b).

### The relationship between plateau pressure and lung strain

It has been recommended that plateau pressure be limited in the practice of protective ventilation. We investigated the relationship between plateau pressure and lung strain. Of note, we found that the correlation (R = 0.301, *p* = 0.007) between the plateau pressure and lung strain was rather low. Whereas all plateau pressures were less than 30 cmH_2_O, 56.3% of patients had lung strain ≥ 0.27.

## Discussion

Low tidal volume ventilation is currently the recommended protocol for ARDS patients. However, a single tidal volume may not be appropriate for all patients [5, 6]. Our study found that respiratory system compliance affected the relationship between tidal volume and strain in ARDS patients. Lung strain did not increase significantly with increasing tidal volumes between 6 and 10 ml/kg PBW in the C_high_ patients. However, among the C_low_ ARDS patients, even with ventilation at a tidal volume of 6 ml/kg PBW, the strain was high enough to induce VILI.

### The limitations of low tidal volume

When strain exceeds the physiological range, excessive expansion or alveolar recruitment and collapse [21] induce VILI [9, 21]. Recently, it was proposed that lung deformation may be one of the key mechanisms of VILI and was therefore defined as a marker to indicate VILI in our study. Our study showed that strain increases gradually with increases in tidal volume and that these parameters are positively correlated. However, in different patients, a given tidal volume will generate different levels of lung strain. Among the patients included in our study, the strain ranged from 0.05 to 1.21 when each patient received mechanical ventilation at a tidal volume of 6 ml/kg PBW.

Furthermore, tidal volume is not the only factor to affect the VILI. Protti and his co-workers reported that a high strain rate, which is the ratio between strain and inspiratory time, is a risk factor for ventilator-induced pulmonary edema [22]. Cressoni et al. also demonstrated that not only tidal volume but also transpulmonary pressure and respiratory rate could induce VILI if they exceed the safe thresholds [23]. Therefore, we need to pay attention to not only tidal volume but also other mechanical factors during mechanical ventilation of patients.

### The limitations of limiting plateau pressure ventilation

Limiting plateau pressure to below 30 cmH_2_O is one key strategy for preventing VILI [24]. Therefore, to avoid the lung injury, we did not increase the tidal volume if the plateau was greater than 30 cmH_2_O. Therefore, some of the strain data at higher tidal volumes were missing for some of the patients. We used the hot-deck imputation to address such missing values to analyze the data.

However, airway plateau pressure is influenced by respiratory system compliance and other factors [25], and there is no clear threshold value that ensures a safe ventilator strategy [7]. For example, the same plateau pressure of 30 cmH_2_O could result in different strains depending on the chest wall elastance. We found that plateau pressure did not correlate with lung strain, possibly as the result of differences in the pleural pressure. Approximately 56.3% of our patients demonstrated a high strain even when the plateau pressure was less than 30 cmH_2_O. This was consistent with findings from previous studies that showed that decreasing the plateau pressure from 29 to 25 cmH_2_O enhanced lung protection in ARDS patients [26]. Our results showed that plateau pressure was not a good index for VILI. In a patient with a fixed compliance, the factor that affects the tidal volume and further affects the strain is the driving pressure not the plateau pressure. Amato et al. demonstrated that decreased driving pressure but not plateau pressure was strongly associated with increased survival. Therefore, setting the tidal volume for an individual based on the driving pressure both in non-ARDS and ARDS patients has been recommended [20, 27].

### Respiratory system compliance affected the effect of tidal volume on driving pressure and strain

Ventilation with a low tidal volume of 6 ml/kg PBW did not improve outcomes for all ARDS patients [5, 6]. One possible reason may be that in patients with a pronounced form of baby lung, ventilation with 6 ml/kg PBW still carries a serious VILI risk because of the high strain. In contrast, among patients with less pronounced baby lung, 6 ml/kg PBW, which generates low strain, could be unnecessarily low, which would increase the risk of supplementary sedation and atelectasis [15]. In our study, we found that the EELV was much higher in patents with high respiratory system compliance (Additional file 2: Figure S1). This result was consistent with those of Rylander and colleagues, which showed that FRC decreased along with respiratory system compliance in ARDS patients [28].

We found that in the patients with low respiratory system compliance, the driving pressure and lung strain could easily exceed the safe thresholds, even when using a tidal volume of 6 ml/kg PBW (Figs. 2 and 3). In addition, our results showed that the strain may exceed the safe range when the tidal volume is increased to 10 ml/kg PBW or higher even in patients with high respiratory system compliance. This result indicated that tidal volumes higher than 10 ml/kg PBW should not be used even in patients with higher respiratory system compliance, which is similar to the recommendations for the use of protective ventilation with lower tidal volumes in non-ARDS patients [29]. Therefore, setting individual tidal volumes based on respiratory system compliance, which is a similar strategy to that based on driving pressure [20], may be a better treatment option.

The change of lung strain with increasing tidal volume depends on the change in EELV. In addition, PEEP is a very important factor that affects the value of EELV. Therefore, the results of the changes of driving pressure and strain were also affected by the PEEP setting rather than purely on the interplay between tidal volume and compliance. In ARDS, a suitable PEEP could recruit the collapsed alveoli, avoid the alveolar overdistension and improve the lung compliance. In contrast, an unsuitable PEEP will decrease the compliance. Therefore, it is important to consider the effect of PEEP when setting the tidal volume during mechanical ventilation.

We found that there was only a relatively low correlation between respiratory system compliance and EELV. However, we found that the EELV was much higher in patents with high respiratory system compliance (Additional file 2: Figure S1). Because the study included only 19 patients, the small sample size could explain the lack of confirmation of a relationship between the compliance and EELV. Interestingly, in the subjects with low compliance and low EELV, we did not find that changes in tidal volume affected the strain more than in the subjects with higher compliance with high EELV. The results showed that the strain decreased more in the C_high_ group than in the C_low_ group when the tidal volume was increased from 6 to 8 ml/kg PBW. There are two possible explanations for this result. First, the conditions of patients with low compliance could have been much more severe than those of the patients with high compliance. According to the results of a previous study, patients with severe disease may have a large amount of recruitable lung and require a higher PEEP [14]. Therefore, when the tidal volume was increased, the mean airway pressure would increase accordingly, which is especially significant in low compliance patients. Furthermore, the increased pressure may recruit the collapsed lung in a gravity-dependent manner and/or induce alveolar overdistension in nondependent areas. These two factors would both increase the EELV and cause smaller changes in the strain as a function of increases in the tidal volume. Second, it is important to note that we did not continue to increase the tidal volume if the plateau exceeded 30 cmH_2_O. For this reason, some of the data for the strain values at higher tidal volumes were missing for some of the patients. We used hot-deck imputation to address any missing values to permit analysis of the data. Therefore, the actual strain may have been higher than we reported. However, we did not know how large the volume would need to be for the differences among the strain values to be significant.

### Driving pressure and lung strain

Driving pressure might be used as a surrogate of lung strain and has been recommended to guide selection of the ventilator settings [20, 27]. We found that the strain increased gradually in parallel with the driving pressure. The strain was significantly higher in the patients with high driving pressures compared to the patients with low driving pressures (Fig. 4a). These results may partly validate the concept of the study of Amato and his colleagues [20]. However, we found only a moderately positive relationship between the driving pressure and the lung strain (Fig. 4b). This result was similar to the relationship between the EELV and respiratory system compliance. It is possible that this result is related to the small sample size. A further study that includes more patients needs to be performed to confirm these results.

Our study had some limitations. First, we did not collect bronchoalveolar lavage fluid from the patients for the measurement of the inflammatory cytokines, so we did not identify a corresponding threshold value for strain in our study. We only showed that there is a risk of exposure to potentially injurious lung strain on the basis of the value of strain that was reported in a previous study. Further study is needed to define a threshold that can indicate VILI.

Second, we calculated respiratory system compliance as the ratio between tidal volume and driving pressure. Actually, compliance is also affected by intra-abdominal pressure, chest wall compliance and other effects [30]. However, our patients had no obvious abdominal hypertension, thoracic deformities or other factors that could affect chest wall compliance. In addition, we defined a respiratory system compliance of 0.6 ml/(cmH_2_O/kg) as the cutoff point for the classification of the patients based on previous studies. Therefore, we believe that these factors had no significant impact on the results.

Third, we calculated only dynamic strain, not static strain, which should also be studied to improve the treatment of ARDS patients. Lung strain is affected by PEEP. With PEEP, lungs are kept tonically inflated above their functional residual capacity, which exposes them to additional static strain [31]. However, PEEP reduces dynamic strain by re-expanding the collapsed lung tissue. In our study, there was no significant difference in PEEP between the two groups, and therefore, PEEP would not be expected to substantially affect our results.

## Conclusions

We concluded that respiratory system compliance affected the relationship between tidal volume and strain in ARDS patients. Using respiratory system compliance could help clinicians to easily recognize subjects at lower or higher risk of being exposed to “safe” or “unsafe” levels of lung strain. Based on these results, we suggest that setting individual tidal volumes should be based on respiratory system compliance and strain. Targeting the tidal volumes based on decreasing driving pressure may be more rational, even in patients with high compliance. Additional studies are required to confirm the usefulness of this ventilation strategy.

## Additional files

Additional file 1: Table S1. Patient hemodynamics during mechanical ventilation at various tidal volumes ($\overline{\mathrm{x}}$ ± s, *n* = 19). (DOC 39 kb)
Additional file 2: Figure S1. The comparison of EELV between patients with high and low respiratory system compliance. ^***^ *p* = 0.0076 for the comparison with patients with low respiratory system compliance. *Abbreviations*: *EELV* end-expiratory lung volume. (TIF 975 kb)
Additional file 3: Figure S2. The comparison of change of strain when tidal volume was increased from 6 to 8 ml/kg PBW in patients with high and low respiratory system compliance. *p* = 0.0002 for the comparison with patients with low respiratory system compliance. (TIF 110 kb)

## Abbreviations

APACHE II: Acute Physiology and Chronic Health Evaluation II
ARDS: Acute respiratory distress syndrome
ARDSp/ARDSexp: ARDS with pulmonary and extrapulmonary origin
EELV: End-expiratory lung volumes
FiO_2_: Fraction of inspired oxygen
FRC: Functional residual capacity
IBW: Ideal body weight
IL: interleukin
PBW: predicted body weight
PEEP: Positive end-expiratory pressure
Pmean: Mean airway pressure
Ppeak: Peak airway pressure
Pplat: Plateau pressure
VILI: Ventilator-induced lung injury
V_T_: tidal volume
