# Supplementary material for: The effects of low tidal ventilation on lung strain correlate with respiratory system compliance
Source: Crit Care. 2017 Feb 3;21:23. doi: 10.1186/s13054-017-1600-x (PMC5291981; doi:10.1186/s13054-017-1600-x)
Supplement: Additional file 1: Table S1. — Patient hemodynamics during mechanical ventilation at various tidal volumes ($\overline{\mathrm{x}}$x¯ ± s, n = 19). (DOC 39 kb) [file 13054_2017_1600_MOESM1_ESM.doc]

Table S1. Patient hemodynamics during mechanical ventilation at various tidal volumes (±s, n=19)

|  |  | V_T_ (ml/kg) |  |  |
| --- | --- | --- | --- | --- |
|  | 6 | 8 | 10 | 12 |
| HR, beats/min |  |  |  |  |
| C ≥ 0.6 (n=9) | 82 ± 15 | 79 ± 18 | 78 ± 17 | 77 ± 19 |
| C<0.6 (n=10) | 106 ± 27 | 101 ± 30 | 110 ± 45 | 81 ± 27 |
| MAP, mmHg |  |  |  |  |
| C ≥ 0.6 (n=9) | 93 ± 16 | 94 ± 17 | 92 ± 18 | 91 ± 17 |
| C < 0.6 (n=10) | 93 ± 19 | 91 ± 18 | 93 ± 24 | 88 ± 35 |
| CVP, mmHg |  |  |  |  |
| C ≥ 0.6 (n=9) | 9.3 ± 3.9 | 9.3 ± 3.9 | 9.4 ± 3.8 | 9.4 ± 4.0 |
| C < 0.6 (n=10) | 12.5 ± 2.5 | 12.1 ± 2.7 | 13.3 ± 3.1 | 12.5 ± 3.5 |

Abbreviations: HR: heart rate; MAP: mean arterial pressure; CVP: central venous pressure; and C: respiratory system compliance.
