# Supplementary figures and images for: The effects of low tidal ventilation on lung strain correlate with respiratory system compliance
Source: Crit Care. 2017 Feb 3;21:23. doi: 10.1186/s13054-017-1600-x (PMC5291981; doi:10.1186/s13054-017-1600-x)

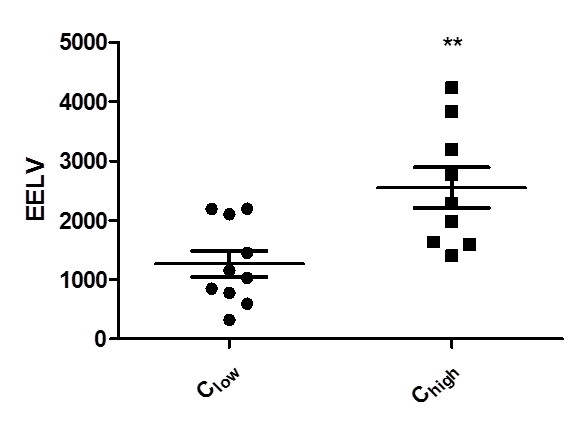

Supplement: Additional file 2: Figure S1. — The comparison of EELV between patients with high and low respiratory system compliance. *** p = 0.0076 for the comparison with patients with low respiratory system compliance. Abbreviations: EELV end-expiratory lung volume. (TIF 975 kb) [file 13054_2017_1600_MOESM2_ESM.tif]
